# Neurological Infection, Kynurenine Pathway, and Parasitic Infection by *Neospora caninum*

**DOI:** 10.3389/fimmu.2021.714248

**Published:** 2022-01-26

**Authors:** Ana Elisa Del’Arco, Deivison Silva Argolo, Gilles Guillemin, Maria de Fátima Dias Costa, Silvia Lima Costa, Alexandre Moraes Pinheiro

**Affiliations:** ^1^ Laboratory of Biochemistry and Veterinary Immunology, Center of Agrarian, Environmental and Biological Sciences, Federal University of Recôncavo of Bahia (UFRB), Cruz das Almas, Brazil; ^2^ Laboratory of Neurochemistry and Cellular Biology, Department of Biochemistry and Biophysics, Institute of Health Sciences, Federal University of Bahia (UFBA), Bahia, Brazil; ^3^ Neuroinflammation Group, Macquarie Medicine School, Macquarie University, Sydney, NSW, Australia

**Keywords:** kynurenic acid, glia, neuroinflammation, *Neospora caninum*, quinolinic acid

## Abstract

Neuroinflammation is one of the most frequently studied topics of neurosciences as it is a common feature in almost all neurological disorders. Although the primary function of neuroinflammation is to protect the nervous system from an insult, the complex and sequential response of activated glial cells can lead to neurological damage. Depending on the type of insults and the time post-insult, the inflammatory response can be neuroprotective, neurotoxic, or, depending on the glial cell types, both. There are multiple pathways activated and many bioactive intermediates are released during neuroinflammation. One of the most common one is the kynurenine pathway, catabolizing tryptophan, which is involved in immune regulation, neuroprotection, and neurotoxicity. Different models have been used to study the kynurenine pathway metabolites to understand their involvements in the development and maintenance of the inflammatory processes triggered by infections. Among them, the parasitic infection *Neospora caninum* could be used as a relevant model to study the role of the kynurenine pathway in the neuroinflammatory response and the subset of cells involved.

## Introduction

As the world population is aging, the rates of diagnosable neurological disorders have increased accordingly, indicating an overall adverse impact on health and quality of life (1). Aiming to understand the etiopathogeneses of an array of neurological disorders, many studies seek to elucidate the potential roles of neuroinflammation (2). Although inflammatory processes may not trigger such disorders alone, the immune system nonetheless can greatly influence symptom severity and progression. Scientists are actively looking for therapeutic targets that may efficiently control the exacerbated immune responses associated with neuroinflammation in such conditions (3, 4).

Neurons and glial cells in the central nervous system (CNS) form complex and coordinated networks, of which a key function is to maintain homeostasis (5). Among glia, both astrocytes and microglia constantly assess the CNS environment for potential insult (6). Astrocytes particularly play key roles in maintaining the integrity of the blood–brain barrier (BBB), regulating CNS metabolism, and releasing antioxidants and trophic factors, as well as participating in the process of synaptic communication (7). On the other hand, microglia are considered the resident immune cells of the CNS, contributing to the pro- and anti-inflammatory immune response as they constantly scrutinize the brain parenchyma to eliminate metabolic waste, abnormal cells and proteins, infectious agents, and damaged tissue (8). In this way, astrocytes and microglia become activated and undergo morphological as well as functional transformations in response to different microenvironmental signals. With the aim to maintain homeostasis, the crosstalk among astrocytes and microglia supports neuronal function and plasticity (9).

Infections, traumatic or ischemic injuries, and accumulation of toxic metabolites often induce dysregulation of brain homeostatic processes. At early stages and/or lower levels of activation, astrocytes and microglia can be neuroprotective (polarized as A2 and M2, respectively) as they initiate coordinated responses to restore homeostasis and limit neurotoxicity by rapidly triggering acute inflammation. This might contribute to tissue repair and neurogenesis, as well as clearance of cellular debris, infectious agents, and abnormal proteins (10, 11). However, at chronic and/or high levels of activation (polarized as A1 and M1), these glial cells can become neurotoxic and contribute to neurodegenerative processes (12). While much has been described, researchers are still trying to fully understand the molecular and cellular triggers for this functional switch (2).

One of the most studied pathways in recent years is the kynurenine pathway (KP), which produces a variety of neuroactive metabolites (13, 14). During neuroinflammation, the KP catabolizes approximately 95% of tryptophan to profoundly decrease serotonin and melatonin production and, instead, generate a host of neurotoxic, neuroprotective, and immune-modulating molecules that play key roles in various brain diseases [reviewed by (15–18)].

Many studies underscore the necessity to better characterize the complex yet coordinated glial response, as well as their relevant communications with neurons in neuroinflammatory-implicated dysfunction (3, 9, 19). In that regard, experimental models able to mimic these interactions may grant opportunities to shed new light on the involvement of tryptophan metabolism in neuroinflammation. For example, exposing external factors such as infectious agents to *in vivo* animal models, *ex vivo* brain tissue slices, and *in vitro* freshly dissociated brain cells can potentially be used to obtain much-needed answers (20). However, effective models should permit recognition by and activation of astrocytes and microglia as well as parasite persistence mechanisms allowing the survival and proliferation of the infectious agents. This immune-escape mechanism might bring information about alternative routes to understand cellular function and linkage of different biochemical pathways, such as KP, and activation and release of neuroprotective factors.

As such, the infection by the parasite *Neospora caninum* appears to be a promising model to study relevant neuroinfectious processes and may contribute to improve the understanding of crosstalk mechanisms between neurons, astrocytes, and microglia. *N. caninum* is an obligate intracellular protozoan, belonging to the phylum *Apicomplexa*, which forms cysts in the CNS and has been shown to lead to abortions in cattle as well as neurological symptoms in dogs (21, 22). Thus, the aim of this review is to highlight the current knowledge about the complex interactions between neuroinflammation and the KP, and to discuss the relevance of the *N. caninum* infection model.

## Neuroinflammation and the Kynurenine Pathway

The KP has been widely studied in the CNS over the last three decades, especially with regard to its interactions with the immune system [reviewed by (23–26)]. That said, the KP is highly dynamic. For instance, many CNS cell types display different KP profiles that depend on the disease and region affected (27–29). Microglial activation rapidly occurs during neuroinflammation and is characterized by structural changes from a putatively resting, surveiling, ramified cell toward an activated, spheroidal one (M1 type) producing proinflammatory mediators such as cytokines (30). These M1 microglia also activate astrocytes, which become reactive themselves (A1 type). Together, these intercellular signals stimulate the release of many proinflammatory mediators, such as cytokines (IL-1β, IL-6, IL-12, IL-23, and TNF-α), chemokine (CCL5 and CCL2), adenosine triphosphate (ATP), reactive oxygen species (ROS), and growth factors (2, 9, 31).

Activation of the KP is associated with induction of the regulatory enzyme, indoleamine 2,3, dioxygenase 1 (IDO-1), and many proinflammatory mediators can stimulate IDO-1 activity. This includes synergistic actions between, for example, TNF-α, IL-1β, and IL-6 (32–34). Other studies have shown that the induction of IDO-1 may also occur in monocyte/macrophage-like cells, even in the absence of IFN-γ (34–36).

After IDO-1, another key step in the KP is the activity of kynurenine mono oxygenase (KMO), an enzyme highly expressed in microglia, which converts kynurenine (Kyn) into 3-hydroxyanthranilic acid (37). The former regulates apoptosis/necrosis pathways in macrophages and has immunoregulatory and T-cell survival properties. Activation of KMO further leads to the formation of quinolinic acid (QA). The best-known action of QA is as an agonist of NMDA receptors in the nervous system (14) and a potent neuro- and gliotoxin (38).

Interestingly, astrocytes do not express KMO while microglia express all enzymatic components of the KP (29, 39, 40). Thus, microglial activation by inflammatory mediators has a fundamental role in increasing the production of QA (41). In homeostatic situations, the production of kynurenic acid (KA) by astrocytes antagonizes, to a certain extent, the excitotoxic effects of the QA produced by the microglia, through its antagonism of NMDA receptors (28). In inflammatory conditions, astrocytes produce large amounts of Kyn that can be taken up and used by microglia as additional substrates to produce QA (29, 42–44). The neurotoxicity of QA is observed through at least five different mechanisms including excitotoxicity by NMDA receptor activation, ROS formation, and cytoskeletal destabilization (45, 46). The imbalance in the production of QA and KA, with accumulation of QA, increases the neurotoxic effects by blocking the glutamate uptake by astrocytes (38). Consequently, this process cyclically stimulates ROS production, disturbs the BBB, and increases phosphorylation of structural proteins such as Tau, neurofilament (NF), and glial fibrillary protein (GFAP), which in turn leads to cellular cytoskeletal destabilization (39, 47, 48).

Together with QA, other catabolites of the KP have synergistic neurotoxic effects. O’Farrell et al. (49) observed a reduced neurite outgrowth and complexity after treatment of neuron cultures with conditioned media derived from BV-2 microglia stimulated with IFN-γ. They also observed an increased concentration of tryptophan, Kyn, and 3-hydroxykynurenine (3-HK) in the conditioned media. When the authors used KP inhibitors, the neuronawfi 2l atrophy was fully prevented.

## Models of Exogenously Activated Neuroinflammation and KP

Many studies have tried to clarify the role of astrocytes and microglia in neuroinflammation and neuroprotection, and each of the mechanisms involved in neuroinflammation are yet to be fully characterized. As above, one common approach to studying neuroinflammation in cell culture and rodent models is exposure to the Gram-negative bacterial lipopolysaccharide (LPS). *In vitro*, LPS induces IFN-γ production and consequently results in the activation of IDO-1 and thus triggers the KP (28, 29, 50, 51). Systemic LPS administration does the same, inducing IDO-1 activity alongside production of brain TNF-α and IL-6 (52).

Despite the effectiveness of stimulating neuroinflammatory processes though, systemic challenge with LPS has failed to fully clarify the mechanisms of KP (53). The response elicited by LPS administration can activate pathogen-associated molecular pattern receptors (PAMPs) and consequently stimulate signaling pathways leading to the production of inflammatory cytokines. Nonetheless, the neuroinflammatory processes triggered in the CNS by infection with bacteria, viruses, and parasites appear to be far more dynamic than the more uniformed responses observed following LPS alone.

On the other hand, there are several models of neuroinfection using microorganisms such as HIV or *Toxoplasma gondii.* The model of infection with *N. caninum* is interesting because it is not infectious to humans, which makes it a safer agent to use in medical research. Furthermore, this parasite is easy to cultivate (54, 55). Importantly, *N. caninum* has the capacity to activate neuroinflammatory processes and grant a viable alternative path to study brain cell interactions and KP activity.

This parasite belongs to the phylum *Apicomplexa*, which are unicellular and spore-forming parasites. Parasites from this group activate the immune response with an associated INF-γ production leading to IDO-1 activation and associated depletion of tryptophan in the host cells. Infection by these groups of parasites also induces an increase in TNF-α and IL-1β production (56, 57). Infection by *Apicomplexa* parasites also triggers an increased production of Kyn, 3-HK, and QA (17, 58).

*N. caninum* infection leads to nervous symptoms in cattle and canids related to infection sites in the CNS [reviewed by (59)]. During its infection, the initial recognition by the immune system involves the toll-like receptors, cytosolic sensors such as nucleotide ligand oligomerization domain-like receptors, and NLR family sensors containing pyrin (60–65). Some studies have shown that the activation of these receptors can lead to an increase of Kyn production *via* the NF-κB signaling pathway (66, 67). During *N. caninum* infection, the lymphocyte T helper 1 (Th1) response is effective to limit the multiplication of the parasite and consequently induces the formation of parasitic cysts in the host. The involvement of lymphocytes T CD4+ and CD8+ is crucial for the development of the anti-parasitic response in mice and is strongly influenced by the systemic increase of IFN-γ (68, 69). Mice treated with recombinant IL-12, which directly is mediated by IFN-γ activity endogenously, had decreased markers of encephalitis as well as brain parasite load 3 weeks later (70). The effectiveness of IFN-γ in protecting against *N. caninum* infection *in vivo* is further supported in a study of mouse strains. Long et al. (71) demonstrated that BALB/c and C57Bl/6 mice were both highly susceptible to the development of *N. caninum*-induced encephalitis, whereas B10.D2 mice were highly resistant. Importantly, splenocytes from B10.D2-infected mice also displayed high antigen-stimulated INF-γ to IL-4 ratios while these ratios were much lower in the other two strains, which indicates that peripheral immune responses favoring INF-γ production might contribute to *N. caninum* protection in select rodent strains *in vivo* (71).

To demonstrate the steps of *N caninum* infection within the CNS, Yamane et al. (72) confirmed that the parasite proliferation in cultured primary bovine brain cells was controlled by IFN-γ as well as TNF-α. Similarly, we have found in mixed cultures of astrocytes and microglia that *N. caninum* induces the production of TNF-α, IL-10, IL-6, and nitric oxide (NO) (55, 73–75). Interestingly, when glia-neuron co-cultures were infected by tachyzoites for 72 h, we observed a *N. caninum*-induced retraction of neurites but no hypothesized neuronal loss (76). However, application of IFN-γ to the medium restored neurite outgrowth of infected cells (76), which is consistent with the IFN-γ-mediated neuroprotection described in the *in vivo* models above. Taken together, we proposed that *N. caninum* infection triggers a local inflammatory response by way of TNF-α and NO production, but without IFN-γ application, the presence of IL-10 and IL-6 may trigger a switch to a Th2 profile, which could help preserve the environment.

Neuro-glia co-cultures infected with *N. caninum* also induced astroglioses, which were characterized by an increased GFAP expression and also induced the mRNA expression for IL-10 (77). At the same time, the infection induced the mRNA expression for brain-derived neurotrophic factor (BDNF) and neuronal growth factor (NGF), which facilitate actions such as synapses plasticity and formation (78). Additionally, treatment of the glia-neuron co-culture with the medium of mixed cultures of astrocytes and microglia infected by this parasite also induced neurite outgrowth (77).

In bovine endothelial cells infected with *N. caninum*, IDO-1 activation was observed in the presence of IFN-γ (79). In a glia-neuron co-culture model, IDO-1 activation was associated with the control of the parasitic proliferation, since inhibiting IDO-1 with 1-MT increased tachyzoite proliferation (80). Also, in the absence of IFN-γ, IDO-1 was activated by the infection, inducing a 50% increase in Kyn compared to uninfected co-cultures. Recently, Argolo et al. (81) demonstrated that, despite observation of neurite outgrowth and release of neurotrophic factors, the infection increased levels of QA and of CCL5 and CCL2 mRNA expression.

These chemokines recruit cells from immune system and activate microglia to control the parasite infection. Aside from its neurotoxic role as an NMDA agonist, QA also contributes to the production of NAD+, which together modulate the production of inflammatory cytokines IL-1β, TNF-α, and IL-6 and facilitate the change toward a pro-inflammatory profile and a resolutive response by the release of IL-10 (29, 82, 83). However, dysregulation of QA production has been seen during infectious models and it is still unclear whether NAD production is altered in these processes. It is possible that a portion of KP metabolites, such as QA, are directed toward NAD+ production in response to infection, but the mechanism(s) mediating this process remain unclear (84).

It is possible that some effects of *N. caninum* infection, such as Th2 cytokine production and release of neurotrophic factors, evidence an atypical immune response associated with parasite persistence. However, these findings should not be confounded with universal neuroprotection as infection progression triggers astrocyte death and neurological impairment. Other studies demonstrated that the parasite could change the immune response to favor its persistence, such as increased population of T CD8+ regulatory cells (68), inhibition of IL-12p40 production (85), and the inhibition of Th1 response by STAT3 phosphorylation in the invasion process (86, 87). Taken together, these varied immune responses to *N. caninum* infection underscore the viability of this model in aiding discovery and further characterization of the dynamic and context-dependent function of neuroinflammatory processes related to the KP and, ultimately, brain function in normal and dysfunctional conditions.

## Conclusion

The KP, the major route of tryptophan catabolism, produces NAD^+^ and several intermediates, which have neuroactive properties. In recent decades, studies of the KP have not only brought new understanding about interactions of these intermediates and the function of CNS, but also highlighted the influence of the unbalanced production of these bioactive catabolites and their potential impact on various neurological disorders. At the same time, many scientists have used *in vitro* experimental models to study cellular mechanisms and pathways involved in both physiological and pathological conditions. The complex and multi-factorial crosstalk between glial cells and neurons have been studied to better understand the processes of brain homeostasis and neuroinflammation, which is a common feature in most brain diseases and disorders.

This paper describes a potentially relevant and novel approach utilizing neuroinfection *via* the parasite *N. caninum* to study KP activation as well as astrocyte/glia crosstalk, particularly given the atypical immune response following infection. Although *N. caninum* triggers an acute inflammatory response marked also by astrogliosis, proinflammatory activation of microglia, and QA production, it also triggers concomitant neurite outgrowth and neurotrophic factor release in culture. This is likely due to the production of neurotrophins and immuno-modulating cytokine IL-10. This highlights the importance of assessing the KP profile and its relationship with other inflammatory molecules in neurological disorders associated with infection by non-LPS factors, such as viruses and parasites, with the aim to understand the consequences of lesser characterized biochemical interactions.

The infectious process most commonly begins in the periphery, resulting in the dysregulation of KP metabolism and alteration of the immune system before propagating in a secondary stage to affect the CNS. The multifactorial and complex interactions between periphery and CNS, the KP, and the alteration of BBB integrity should all be taken into consideration when using models of neuroinfection. The systematic and comprehensive characterization of this response presents another step toward a better understanding of cellular and molecular communication mechanisms between all the protagonists and the inflammatory response triggered by the parasitic infection. With this concluded, some questions remain unanswered: (1) how the mechanism of neural protection occurs, even with the increase in QA, and whether this relationship may bring new insights to understand the CNS response to external insult; (2) how CNS homeostasis is disrupted after a systemic challenge with *N. caninum*, where the KP intermediates are produced, and by which brain cells; (3) what other metabolic pathways would be associated with KP to justify a neuroprotective response; (4) do the atypical immune responses described in cell culture studies translate fully to *in vivo* models or provide additional novelty for understanding relevant immune activity; and (5) can the *N. caninum* infection model revolutionize our understanding of the cellular crosstalk in the CNS, highlight new processes worthy of investigation, and ultimately facilitate the development of more effective therapeutic interventions for immune-related dysfunction of the brain.

## Author Contributions

AE, MF, and AP performed the literature search and drafted the manuscript. DA wrote a section of the manuscript. MF, GG, SC, and AP contributed to the exchange of knowledge, and wrote, edited, and critically revised the manuscript. All authors contributed to the article and approved the submitted version.

## Funding

This work was supported by the National Council for Scientific and Technological Development (CNPq, Process 431927/2016-2; INCT) and the Foundation for Research Support of the State of Bahia (FAPESB, Processes APP 0058/2015). GG is funded by the NHMRC.

## Conflict of Interest

The authors declare that the research was conducted in the absence of any commercial or financial relationships that could be construed as a potential conflict of interest.

## Publisher’s Note

All claims expressed in this article are solely those of the authors and do not necessarily represent those of their affiliated organizations, or those of the publisher, the editors and the reviewers. Any product that may be evaluated in this article, or claim that may be made by its manufacturer, is not guaranteed or endorsed by the publisher.
